# Cell‐free DNA for detection and monitoring of extramedullary AML relapse

**DOI:** 10.1002/hem3.70097

**Published:** 2025-03-10

**Authors:** Henri C. Hupe, Clara P. Wienecke, Stephan Bartels, Elisa Schipper, Jannika Leßmann, Alina Lasch, Maximilian Bader, Razif Gabdoulline, Martin Neugebohren, Elke Dammann, Hans H. Kreipe, Ulrich Lehmann, Anke K. Bergmann, Nataliya Di Donato, Michael Stadler, Matthias Eder, Arnold Ganser, Florian H. Heidel, Felicitas Thol, Michael Heuser

**Affiliations:** ^1^ Department of Hematology, Hemostasis, Oncology and Stem Cell Transplantation Hannover Medical School Hannover Germany; ^2^ Institute of Pathology Hannover Medical School Hannover Germany; ^3^ Department of Human Genetics Hannover Medical School Hannover Germany; ^4^ Cellular Therapy Center (CTC) Hannover Medical School Hannover Germany; ^5^ Leibniz Institute on Aging Fritz‐Lipmann‐Institute Jena Germany; ^6^ Department of Internal Medicine IV University Hospital Halle (Saale), Martin‐Luther‐University Halle‐Wittenberg Halle Germany

## Abstract

Isolated extramedullary manifestations (IEM) of acute myeloid leukemia (AML) are recurrent events, especially following allogeneic hematopoietic cell transplantation (alloHCT). To date, measurable residual disease (MRD) assessment for this difficult‐to‐treat patient cohort has not been established. In this study, we evaluated highly sensitive next‐generation sequencing (NGS) of IEM‐AML tumor and compared it with cell‐free DNA (cfDNA) from plasma, as well as highly sensitive NGS analysis of bone marrow mononuclear cells (BMMC) and peripheral blood mononuclear cells (PBMC), in a cohort of 15 IEM‐AML patients with 19 IEM‐AML episodes. cfDNA demonstrated a superior representation of IEM‐AML tumor mutations compared to BMMC or PBMC, with a median variant allele frequency (VAF) of 0.8% and a mutation detection rate of 62% (37 of 60 mutations), compared to a median VAF of 0.05% and detection rate of 27%, respectively (16 of 60 mutations, *p* < 0.01). Among 44 mutations identified in 14 IEM‐AML relapse tumors, 30 mutations (68%) were known from initial diagnosis. Using diagnostic mutations from initial diagnosis for MRD analysis and detection of IEM‐AML relapse, 16 of 17 IEM‐AML relapse episodes were detected via cfDNA, whereas only 7 of 17 were identified using conventional analysis of BMMC or PBMC. Our findings demonstrate that cfDNA analysis from plasma effectively captures the molecular profile of IEM‐AML. More than one‐third of clinically relevant mutations were exclusively detected through cfDNA and were missed by conventional NGS‐MRD of BMMC or PBMC. These results suggest that MRD monitoring using cfDNA offers a more comprehensive and sensitive approach to detecting IEM‐AML relapse compared to standard methods.

## Graphical abstract

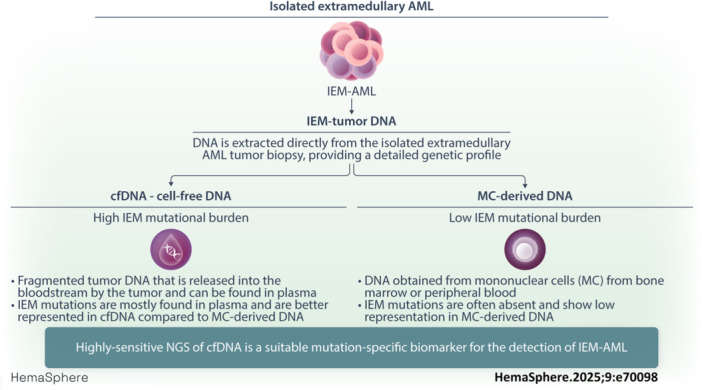

## INTRODUCTION

Extramedullary acute myeloid leukemia (EM‐AML), also known as myelosarcoma, is a rare and clinically severe form of AML, characterized by the presence of leukemic cells outside the bone marrow (BM) and peripheral blood (PB). EM‐AML can present either as an isolated extramedullary occurrence or in combination with medullary involvement. Isolated extramedullary AML (IEM‐AML) is particularly challenging as it often evades detection by conventional diagnostic methods, necessitating the use of advanced imaging, histopathology, and molecular techniques for accurate identification and characterization.

IEM‐AML most commonly occurs as a relapse following allogeneic hematopoietic cell transplantation (alloHCT).^1^, ^2^ The outcome of IEM‐AML relapse appears to be more favorable compared to medullary relapse post‐alloHCT.^3^, ^4^ However, the overall prognosis for patients with IEM‐AML relapse remains poor, due to the challenges in achieving long‐term remission with standard treatment approaches. In contrast to medullary relapse, graft‐versus‐host disease does not appear to provide protection against IEM‐AML relapse.^5^, ^6^, ^7^ The lack of reliable tools to identify the occurrence and the therapeutic response further complicates the clinical management of IEM‐AML.

Recent advancements in liquid biopsy technologies, particularly the analysis of cell‐free DNA (cfDNA) or circulating tumor DNA (ctDNA) in solid oncology,^8^, ^9^, ^10^ offer new possibilities for the detection and monitoring of IEM‐AML. While cfDNA is present at low concentrations in healthy individuals, its levels can rise significantly in various pathological conditions, such as systemic inflammation, autoimmune disorders, trauma, and particularly in malignancies.^11^ Cell‐free DNA is predominantly released into the bloodstream through apoptosis and necrosis, although active secretion by living cells has also been postulated.^11^, ^12^, ^13^, ^14^ The majority of cfDNA is derived from hematopoietic cells.^15^, ^16^, ^17^ Typically, cfDNA fragments measure approximately 150–200 base pairs, but in cancer and other pathological conditions, their size can vary more widely, reflecting abnormal cellular turnover.^14^ Due to its relatively short half‐life, ranging from as little as 16 min to a few hours,^18^, ^19^ cfDNA offers a real‐time reflection of pathological processes, accurately capturing changes in the mutational landscape during disease progression.^17^, ^20^

The prognostic value of ctDNA analysis has been well established in several solid tumors and hematologic malignancies.^17^, ^21^, ^22^, ^23^, ^24^ For example, in stage II colon cancer, ctDNA detection has been shown to predict a higher risk of relapse and helps refine adjuvant chemotherapy decisions.^10^, ^25^ Similarly, in lymphomas, particularly in diffuse large B‐cell lymphoma, baseline ctDNA levels and ctDNA molecular responses have been identified as independent prognostic markers.^26^, ^27^, ^28^

In the setting of AML and myelodysplastic syndrome, ctDNA analysis following alloHCT has been shown to be highly predictive of relapse, surpassing DNA analysis from PB mononuclear cells (MC). Furthermore, there is evidence that cfDNA may offer greater sensitivity in detecting small clones compared to bone marrow (BM)‐derived DNA.^17^, ^20^

As IEM‐AML shows characteristics of a solid tumor, we hypothesized that cfDNA correlates with the cell mass and clinical activity of IEM‐AML.

Thus, we designed a retrospective study to explore the utility of cfDNA analysis in the context of IEM‐AML. We aimed to: (1) investigate whether IEM‐AML can be detected using NGS of cfDNA, (2) understand the relative abundance of leukemic cells in different tissues by comparing cfDNA analysis with IEM‐AML tumor and BM or PBMC, and (3) evaluate cfDNA analysis as a tool for monitoring IEM‐AML patients.

## PATIENTS, MATERIALS, AND METHODS

Patients were eligible for retrospective cfDNA analysis if they were 18 years of age or older, had a diagnosis of AML as defined by the 2022 ICC,^29^ a histologically proven isolated extramedullary manifestation of AML without medullary involvement (BM blast count < 5%), and were treated at our institution between 2008 and 2024.

A total of 769 patients from our institution were screened for an EM‐AML. One hundred and forty‐one patients were identified with an EM‐AML, of which 37 had an IEM‐AML. Twelve patients were excluded due to the lack of available cfDNA samples, and ten additional patients with isolated central nervous system (CNS) relapse were also excluded. Eight patients developed a second IEM‐AML manifestation during the course of their disease, and paired samples from cfDNA and PB/BMMC were available for analysis in four of these cases. Overall, our analysis included 19 IEM‐AML episodes (Supporting Information S1: Figure S1).

### Molecular and cytogenetic analysis

For molecular analysis, DNA from ficoll‐separated BM or PB samples was extracted using the Allprep DNA/RNA purification kit, and cfDNA from plasma was extracted using the QIAamp Circulating Nucleic Acid Kit (Qiagen). Due to the retrospective nature of the analysis, STRECK tubes were only implemented starting in 2021. Prior to that, plasma was centrifuged immediately after collection and frozen in standard monovette tubes. DNA from whole blood cell pellets was extracted using the DNeasy Blood and Tissue Kit (Qiagen). DNA extraction from tumor tissue was carried out using the Maxwell RSC instrument (Promega) following the manufacturer's instructions. Depending on the size of the trephine sample, three to five sections of 10 µm thickness were taken and DNA was extracted using the Maxwell RSC DNA FFPE kit.^16^

For MC‐DNA analysis, we utilized BM samples at the time of IEM‐AML diagnosis in 16 of the 19 IEM‐AML episodes; PB samples were used in cases where BM samples were unavailable. Blast cells from diagnosis and relapse were analyzed for recurrently occurring mutations in AML patients with a custom TruSight myeloid panel covering 46 AML‐related entire genes or hotspots (Illumina) (Supporting Information S1: Table S1),^30^, ^31^ or the Oncomine myeloid panel covering 40 AML‐related entire genes or hotspots for myelosarcoma tissues (ThermoFisher).^32^ For NGS analysis, we used an amplicon sequencing approach for the highly sensitive detection of SNVs and indels (limit of detection 0.01%), which significantly reduces sequencing error rates, as previously described.^30^ DNA library preparation of cfDNA samples involved using a proofreading high‐fidelity polymerase for all PCRs, performing twenty cycles for the first PCR, employing random barcodes as read family tags for in silico error‐correction, and utilizing multiplex identifiers (MIDs) uniquely combined within each library. A minimum of 10 ng absolute input DNA was required per marker. Sequencing was done on the MiSeq® instrument using the Illumina MiSeq reagent kit v3 (600 cycles) with reads covering 251 bases in both forward and reverse directions.

Cytogenetic analysis was performed centrally by G‐ and R‐banding analysis and was described according to the International System for Human Cytogenetic Nomenclature (ISCN 2020).^33^

### Bioinformatic analysis and error‐corrected sequencing of patient‐specific mutations

Bioinformatic analysis of myeloid panel and amplicon‐based error‐corrected sequencing was conducted as previously described.^30^ Sequencing reads were aligned to the human genome hg19 using BWA software (Burrows‐Wheeler Aligner, algorithm option ‘mem,’ ‘paired‐end’). The presence of *FLT3*‐ITDs or insertions was tested with various methods to enhance sensitivity and specificity. Labeling a variant as MRD positive or negative followed a standardized algorithm as previously described.^30^ This algorithm differentiates between single nucleotide variants (SNVs), small or large indels based on the number of read families (RF mode, error‐corrected sequencing) or the number of matching forward (R1) and reverse (R2) reads (R1/R2 mode) to establish the limit of detection (LOD). The LOD for SNVs and small indels is defined as the average background error plus three standard deviations of the background error. This error is calculated using the largest non‐reference variant allele frequency (LVAF) between the primers of the respective amplicon. A large indel of more than three base pairs is considered positive if supported by ≥75 mutated reads, except for the *NPM1* four‐nucleotide insertion, where the requirement is ≥10 supporting reads. The presence of *FLT3*‐ITDs or insertions was tested with two other methods, getITD and FiLT3r,^34^, ^35^ and considered positive if ≥50 supporting reads were found.

All mutations that were detected in the IEM‐AML relapse samples were monitored for MRD, and the patient was defined as cfDNA or BM/PBMC positive if at least one of the mutations was positive.

If a relapse mutation was detectable in both the diagnostic sample and in the IEM‐AML relapse tumor tissue, it was considered ‘stable.’ If a mutation was detected only at diagnosis but not at relapse, it was considered ‘lost,’ and if it appeared only at IEM‐AML relapse, it was considered ‘gained’.

### Statistical analysis

The statistical analysis was performed with the statistical software package SPSS 26.0 (IBM Corporation), GraphPad Prism (Version 9.3.1), statistical program R and RStudio, and Microsoft Excel 2019, version 16.64 (Microsoft Corporation, Redmond, WA). Sankey diagrams were performed using SankeyMATIC (Version 2023‐11‐11).

## RESULTS

### Patient characteristics

Of the 15 included patients with IEM‐AML, eleven were male and four were female (see Table 1 for patient characteristics). All patients received intensive induction and consolidation chemotherapy. Six patients had an adverse ELN 2022 risk, while nine had a favorable or intermediate ELN 2022 risk.^36^ Four patients had secondary AML, and four exhibited a complex karyotype. Thirteen patients developed an IEM‐AML relapse post alloHCT, one patient had IEM‐AML after intensive chemotherapy, and one presented with an IEM‐AML at initial diagnosis. At the time of IEM‐AML manifestation, median blood counts were within the normal range. The median time from alloHCT to IEM‐AML relapse was 449 days. Eight patients developed a second IEM‐AML episode during the course of their disease, of which four could be analyzed. The most common sites of IEM‐AML were the skin or mucosa and the musculoskeletal system (Table 1). Only one patient of our cohort with *FLT3‐ITD* positive disease received a targeted therapy with gilteritinib in combination with radiotherapy at the time of IEM‐AML relapse, achieving CR before undergoing transplantation (Supporting Information S1: Figure S6). Other patients with *FLT3‐ITD* (*n* = 1), *FLT3‐TKD* (*n* = 3), and *atypical FLT3* mutations (*n* = 1) did not receive FLT3 inhibitors, as these therapies were not available at the time of diagnosis and relapse. None of the patients in our cohort received IDH inhibitor therapy (Table 1, Supporting Information S1: Figure S6).

**Table 1:** Clinical, molecular, and transplantation‐associated characteristics in 15 patients with IEM‐AML.

Characteristics	All patients (*n* = 15)
Median age (range)	56 (18–74)
Sex, *n* (%)	
Male	11 (73)
Female	4 (27)
Intensive therapy, *n* (%)	
Yes	15 (100)
No	0 (0)
ECOG performance status, *n* (%)	
0	8 (53)
1	6 (40)
2	1 (7)
Type of AML, *n* (%)	
De novo	11 (73)
Secondary	4 (27)
Therapy‐related	0 (0)
2022 ICC classification, *n* (%)	
AML with recurrent genetic abnormality	6 (40)
AML with MRGM	3 (20)
AML with MRCA	3 (20)
AML not otherwise specified (NOS)	2 (13)
AML with mutated TP53	0
Myeloid sarcoma	1 (7)
ICC 2022 diagnostic qualifiers, *n* (%)	
De novo	11 (73)
Therapy‐related	0
Progressed from MDS	4 (27)
Progressed from MDS/MPN	0
Germline predisposition	0
2022 ELN risk classification, *n* (%)	
Favorable	4 (27)
Intermediate	5 (33)
Adverse	6 (40)
Cytogenetic risk group, *n* (%)^a^	
Favorable	2 (13)
Intermediate	9 (60)
Adverse	4 (27)
Complex karyotype, *n* (%)	
No	11 (73)
Yes	4 (27)
Prior history of solid tumor, *n* (%)	
No	15 (100)
Yes	0 (0)
IEM‐WBC count, ×10^9^/L	
Median	6.8
Range	3.4–15.2
IEM‐hemoglobin, g/dL	
Median	13.8
Range	10.5–16.1
IEM‐platelet count, ×10^9^/L	
Median	183
Range	81–473
Isolated extramedullary relapse post‐alloHCT, *n* (%)	
No	2 (13)
Yes	13 (87)
Second extramedullary relapse, *n* (%)	
No	7 (47)
Yes	8 (53)
Extramedullary site, *n* (%)^b^	
Skin/mucosa	6 (40)
Musculoskeletal	6 (40)
Orbita	3 (20)
Kidney	3 (20)
Lymphatic tissue	3 (20)
Breast	1 (7)
Bladder	1 (7)
Anterior mediastinum	1 (7)
Treatment of IEM‐AML, *n* (%)^c^	
Radiotherapy	10 (67)
Intensive chemotherapy	7 (47)
DLI	6 (40)
AlloHCT	4 (27)
Non‐intensive chemotherapy	2 (13)
*FLT3*‐Inhibition	1 (7)
AlloHCT at any timepoint, *n* (%)	
No	1 (7)
Yes	14 (93)
AlloHCT as first‐line therapy, *n* (%)	
No	5 (33%)
Yes	10 (67%)
Median days alloHCT to IEM‐AML (range) (*n* = 13)	449 (190–3301)
Donor match, *n* (%)	
MR	4 (31)
MU	7 (54)
MMR	0 (0)
MMU	2 (14)
Type of conditioning regimen, *n* (%)	
Myeloablative	4 (31)
Reduced intensity	9 (69)
Remission status pre‐alloHCT, *n* (%)	
CR1	4 (30)
CRi1	1 (8)
CR2	3 (23)
CRi2	1 (8)
PR	1 (8)
Refractory	3 (23)
HCT‐CI score	
<3	6 (46)
≥3	4 (31)
Missing	3 (23)
Stem cell source, *n* (%)	
PB	12 (92)
BM	1 (85)

Abbreviations: AlloHCT, allogeneic hematopoietic cell transplantation; BM, bone marrow; CR, complete remission; DLI, donor lymphocyte infusion; ECOG, Eastern Cooperative Oncology Group; ELN, European LeukemiaNet; HCT‐CI, Hematopoietic Cell Transplantation‐Comorbidity Index; ICC, International Consensus Classification; IEM, isolated extramedullary; MMR, mismatched related; MMU, mismatched unrelated donor; MR, matched related; MRCA, myelodysplasia related cytogenetic abnormalities; MRGM, myelodysplasia‐related gene mutations; MU, matched unrelated; NA, not available; PB, peripheral blood; PR, partial remission; WBC, white blood count.

^a^ Cytogenetic risk group is defined according to the Medical Research Council criteria.^37^

^b^ Multiple sites per patient, hence percentages exceed 100%.

^c^ Multiple therapies per patient, hence percentages exceed 100%.

The most commonly used imaging modality for the detection of the first IEM‐AML was CT (8 of 15 patients, 53%), followed by MRI (5 of 15 patients, 33%) and PET/CT (2 of 15 patients, 13%) (Supporting Information S1: Table S3). For follow‐up, most patients with a visible tumor were monitored by clinical examination (8 of 19 IEM‐AML episodes, 42%).

### IEM‐AML relapse mutations are represented in cfDNA

**Figure 1:**
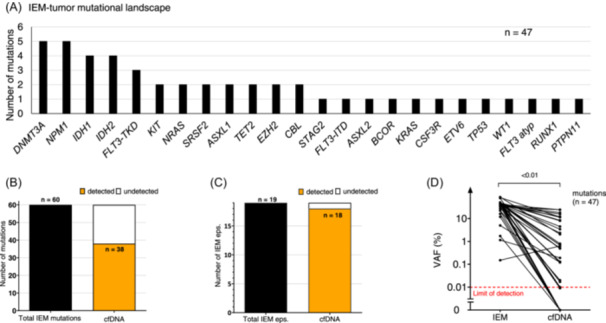
**IEM‐AML relapse mutations are represented in cfDNA**. **(A)** Frequency of genes that were mutated in the IEM‐AML tumor tissue of 15 patients was assessed by highly sensitive NGS. **(B)** Ratio of IEM‐AML mutations and **(C)** IEM‐AML episodes that were detected by NGS analysis of cfDNA using the mutations from IEM‐AML tumor tissue. **(D)** Comparison of VAF (%) between IEM‐AML tumor and cfDNA. atyp, atypical; cfDNA, cell‐free DNA; eps., episodes; IEM, isolated extramedullary AML; inv, inversion; ITD, internal tandem duplication; TKD, tyrosine kinase domain; VAF, variant allele frequency.

First, we sequenced the IEM‐AML tumor tissue using our NGS‐based myeloid panel. A total of 47 mutations were detected in the 15 IEM‐AML tumor manifestations, with a median of three mutations per IEM‐AML patient (range 1–6). The most frequently mutated genes were *DNMT3A* (*n* = 5), *NPM1* (*n* = 5), *IDH1/2* (both *n* = 4), and *FLT3‐*TKD (*n* = 3) (Figure 1A, Supporting Information S1: Table S2). For two of the second IEM‐AML episodes, tumor tissue was available, while for the other two, we relied on the known mutations from the first IEM‐AML tumor tissue. In total, we analyzed 60 mutations across all 19 IEM‐AML episodes. Next, we analyzed how many of these mutations are represented in cfDNA from plasma using our highly sensitive NGS approach. The median amount of input DNA used per marker was 20.1 ng (range 10–51.5 ng). *DNMT3A*, *TET2*, and *ASXL1* (DTA) mutations are frequently associated with clonal hematopoiesis of indeterminate potential (CHIP) and were found in 7 of 15 patients (47.7%). As previously shown,^38^ we were able to use these mutations as MRD markers post‐alloHCT. Thirty‐seven of 60 (62%) IEM‐AML tumor mutations were detectable in cfDNA (Figure 1B). This allowed the detection of 18 out of 19 IEM‐AML episodes (95%) using cfDNA (Figure 1C). The variant allele frequency (VAF) was significantly higher in the IEM‐AML tumor compared to cfDNA from plasma with a median VAF of 45% (range 0.15%–90%) compared to 0.88% (range 0.017%–24.2%) (Figure 1D). Only the VAF of one *NPM1*‐mutation was higher in cfDNA compared to the IEM‐AML tumor (0.63 vs. 0.15%). No significant correlation was observed between the VAF of mutations in the IEM‐AML tumor and those detected in cfDNA (Supporting Information S1: Figure S2A). Additionally, the VAF in the IEM‐AML tumor did not differ significantly between mutations that were detected in plasma and those that were not (45% vs. 42%, *p* = 0.123, Supporting Information S1: Figure S2B). Thus, IEM‐AML relapse mutations are well represented in cfDNA when analyzed with a highly sensitive detection method, although we found no direct correlation between the mutation burden of the IEM‐AML tumor and the mutation burden in plasma.

### IEM‐AML relapse mutations are better represented in cfDNA compared to MC‐derived DNA

**Figure 2:**
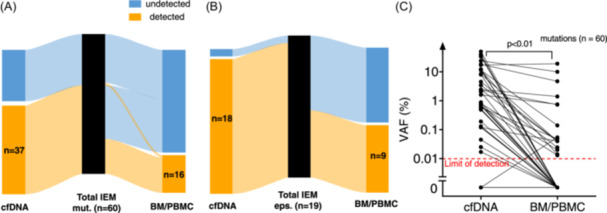
**Comparison of cfDNA versus MC‐derived DNA for detection of IEM‐AML. (A)** Number of detected IEM‐AML mutations and **(B)** number of IEM‐AML episodes in the cfDNA and BM/PBMC compartments. **(C)** VAF comparison of matched mutations between cfDNA and BM/PBMC‐derived DNA for all IEM‐AML episodes. BM, bone marrow; cfDNA, cell‐free DNA; IEM, isolated extramedullary AML; eps, episodes; mut, mutations; MC, mononuclear cells; mut., mutated; PB, peripheral blood; VAF, variant allele frequency.

When performing conventional NGS analysis of BM (*n* = 16) or PBMC (*n* = 3) derived DNA at the time of the IEM‐AML manifestations, only 16 of the 60 (27%) IEM‐AML mutations were detected in MC‐derived DNA (Figure 2A). In the analysis of 19 IEM‐AML episodes, only nine (47%) were identified by conventional BM/PBMC analysis (Figure 2B). Notably, one mutation (*CBL*, VAF 0.05%) was detected exclusively in BM and missed in cfDNA. The VAF of mutations was significantly higher in cfDNA compared to BM/PBMC DNA with a median VAF of 0.88% (range 0.17%–24.2%) in cfDNA versus 0.05% (range 0.013%–19.05%) in BM/PBMC DNA (Figure 2C). This indicates that the IEM‐AML clones are most abundant in the IEM‐AML tumor, followed by cfDNA, and least abundant in BM/PBMC‐derived DNA. Thus, IEM‐AML relapse mutations are better represented in cfDNA compared to MC‐derived DNA, thereby confirming that mutated cfDNA derives from IEM‐AML tissue and not PB or BMMC.

There was no significant difference in the cfDNA detection rate between IEM‐AML episodes with a single lesion and those with multiple lesions. In 8 of 19 IEM‐AML episodes, multiple localizations were observed across the same or different tissues. cfDNA detected 7 of 8 episodes (88%) and 16 of 22 mutations (73%). For cases with a single localization, cfDNA detected all 11 episodes (100%) and 21 of 38 mutations (55%) (*p* = 0.18, comparing detected mutations between single and multiple localizations). In contrast, BM/PBMC evaluation identified only 3 of 8 episodes (38%) and 7 of 22 mutations (32%) in cases with multiple lesions, and 6 of 11 episodes (55%) and 9 of 38 mutations (24%) in cases with a single localization (*p* = 0.49). A key limitation of this analysis is the variability in diagnostic modalities used at the time of IEM‐AML diagnosis (Supporting Information S1: Table S3), which may have resulted in undetected lesions and an underestimation of patients with multiple IEM‐AML localizations. We also evaluated the detection rate of mutations in cfDNA compared to BM/PBMC across different organ involvements (Supporting Information S1: Figure S7). cfDNA consistently demonstrated higher detection rates across all organ involvements compared to BM/PBMC analysis.

### IEM‐AML relapse can be well detected using diagnostic mutations for MRD monitoring

**Figure 3:**
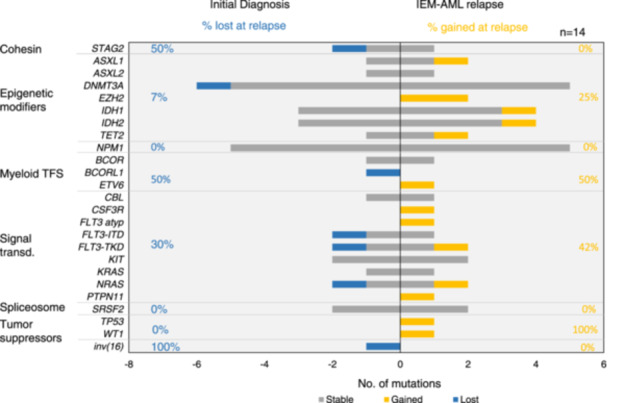
**Clonal evolution of mutations from the initial diagnosis to IEM‐AML relapse.** Number of mutations lost and gained at IEM‐AML relapse sorted by mutation classes, from 14 AML patients who developed an IEM‐AML relapse. Thrity of 44 (68%) mutations found in the IEM‐AML relapse tumor were known from the initial diagnosis. atyp, atypical; IEM, isolated extramedullary AML; Inv, inversion; ITD, internal tandem duplication; no., number; TFS, transcription factors; transd, transduction; TKD, tyrosine kinase domain.

To compare the clonal evolution from diagnosis to IEM‐AML we compared the mutational landscape in the 14 patients who developed IEM‐AML relapse (Figure 3 **)**. One patient with an IEM‐AML at initial diagnosis without IEM‐AML relapse was excluded from this analysis. Of a total of 49 mutations that were detected at diagnosis and/or relapse, 30 (61%) were present at both timepoints. However, 14 mutations (29%) were gained and seven mutations (14%) were lost at first IEM‐AML relapse (Figure 3, Supporting Information S1: Figure S3). Thirty of 44 (68%) IEM‐AML relapse mutations were known from initial diagnosis. Mutations in epigenetic modifiers (14 of 21, 67%), *NPM1* (5 of 5, 100%), and *SRSF2* (2 of 2, 100%) appear to be more stable in IEM‐AML relapse, while mutations in signal transduction pathways (7 of 15, 47%), myeloid transcription factors (1 of 3, 33%), and tumor suppressor genes (0 of 2, 0%) were relatively unstable and were most frequently lost or acquired at relapse.

We next evaluated how IEM‐AML relapses would be represented in plasma and BM/PBMC if only the mutations known from the initial diagnosis were used. One patient with IEM‐AML at initial diagnosis and another without a known molecular marker from initial diagnosis were excluded from this analysis. Using the 41 mutations from the initial diagnosis, 16 of 17 (94.1%) IEM‐AML episodes and 29 of 41 (71%) mutations were detected in cfDNA from plasma at the time of IEM‐AML relapse (Supporting Information S1: Figure S4). However, only 7 (41%) IEM‐AML episodes and 13 (32%) mutations were detected by conventional analysis of MC‐derived DNA at the time of IEM‐AML relapse. The mutation detection rate in cfDNA at IEM‐AML relapse was higher for stable mutations (27 of 38, 71%) compared to those gained at relapse (10 of 24, 42%, *p* = 0.033) (Supporting Information S1: Figure S5A), suggesting that the known mutations from initial diagnosis are reliable markers for monitoring IEM‐AML relapse. However, the median VAF of mutations detected in cfDNA did not significantly differ between mutations acquired at IEM‐AML relapse and those that remained stable (Supporting Information S1: Figure S5B).

### Cell‐free DNA to monitor treatment response of IEM‐AML

**Figure 4:**
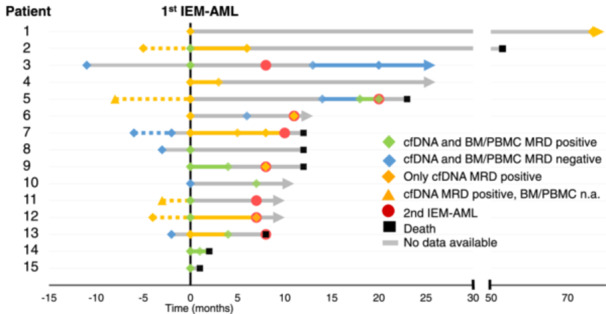
**Longitudinal NGS‐MRD analysis of mutations from IEM‐AML tumors using cfDNA and BM/PBMC.** X‐axis: Time before (negative values) and after (positive values) the first IEM‐AML episode (First IEM‐AML = 0). *Y*‐axis: Number of patients included in our retrospective analysis. Green diamond and line: Both cfDNA and BM/PBMC‐derived DNA analysis positive. Blue diamond and line: Both cfDNA and BM/PBMC‐derived DNA analysis negative. Yellow diamond: cfDNA analysis positive, BM/PBMC‐derived DNA analysis negative. Yellow triangle: cfDNA analysis positive, BM/PBMC sample not available. Yellow line: cfDNA analysis positive. Red circle: Second IEM‐AML during the course of the disease. Black rectangle: Patient deceased. Triangle at the end: Patient still alive at last follow‐up. Gray line: No data available at that time point. Dashed lines: MRD‐status before first IEM‐AML. Solid lines: MRD‐status after 1st IEM‐AML. BM, bone marrow; cfDNA, cell‐free DNA; IEM, isolated extramedullary; MRD, measurable residual disease; N/A, not available; PB, peripheral blood.

We next evaluated whether cfDNA can be used for MRD monitoring before IEM‐AML relapse and during the course of treatment (Figure 4, Supporting Information S1: Figure S6, Table S2).

Due to the retrospective nature of this analysis, cfDNA samples before IEM‐AML diagnosis were available for eight patients. Four of eight patients were MRD positive in cfDNA at eight, five, four, and three months before IEM‐AML relapse. For two of these cfDNA MRD‐positive cases, matched PBMC samples were available and MRD analysis from MC‐derived DNA was negative. Four of eight IEM‐AML patients were MRD‐negative in both compartments at eleven, six, three, and two months before IEM‐AML relapse (Figure 4, Supporting Information S1: Figure S6).

For ten patients, cfDNA and matched BM/PBMC samples were available during or after IEM‐AML treatment (Figure 4, Supporting Information S1: Figure S6). The treatments varied among patients (Table 1, Supporting Information S1: Figure S6).

Four patients experienced progressive disease (PD) before completing IEM‐AML therapy. Two patients (Patients 14 and 4) were cfDNA MRD‐positive at one and three months and subsequently developed PD three and four months after their first IEM‐AML diagnosis, respectively. Two patients (Patients 15 and 11) had no available MRD samples and exhibited PD at 1 and 9 months after IEM‐AML diagnosis (Figure 4, Supporting Information S1: Figure S6).

Two patients (Patients 9 and 13) achieved partial remission (PR) during therapy and were MRD‐positive in both compartments at 3 months post IEM‐AML and before alloHCT. Both of them relapsed with a second IEM‐AML at 3 and 5 months after alloHCT. Patient 12, for whom no MRD samples were available, achieved PR after donor lymphocyte infusion (DLI) and relapsed 4 months later with a second IEM‐AML following additional radiotherapy (Figure 4, Supporting Information S1: Figure S6).

Three patients (Patients 2, 3, and 7) achieved PR after completing IEM‐AML therapy. Patient 2 was cfDNA‐positive at 6 months after IEM‐AML diagnosis and shortly after DLI infusion, and deceased later due to pulmonary graft‐versus‐host disease without signs of IEM‐AML relapse. Patient 3 was MRD‐negative at 12 months post‐IEM‐AML in both compartments and showed no signs of relapse for up to 30 months post‐IEM‐AML relapse. Patient 7, who was MRD‐positive at 6 months after IEM‐AML and after treatment with high‐dose cytarabine and radiotherapy, relapsed 5 months later with IEM‐AML (Figure 4, Supporting Information S1: Figure S6).

Four patients achieved complete remission (CR) after completing IEM‐AML therapy. Two of them (Patients 5 and 6) were cfDNA‐negative at 12 and 6 months post‐IEM‐AML but relapsed 12 and 6 months after achievement of cfDNA MRD‐negative CR. For two patients in CR (Patients 1 and 10), no follow‐up samples were available during remission (Figure 4, Supporting Information S1: Figure S6).

Lastly, Patient 8 did not undergo any follow‐up screening, and no samples were available for MRD evaluation in the retrospective analysis.

For four of eight patients matched samples from cfDNA and BM/PB were available for the time of the second IEM‐AML episode. Of these, three were positive only in the cfDNA compartment but not in BM/PBMC‐derived DNA (Figure 4, Supporting Information S1: Figure S6).

Thus, longitudinal MRD assessment provides valuable additional information to imaging techniques, offering a promising approach for evaluating treatment response and identifying IEM‐AML patients at risk of second relapse by monitoring known mutations in cfDNA from plasma (Figure 4, Supporting Information S1: Figure S6).

## DISCUSSION

In this study, we evaluated the molecular representation of IEM‐AML across different compartments, including the respective extramedullary tissue, plasma, and MC from PB and BM. Our analysis demonstrates that cfDNA analysis is highly sensitive for detecting IEM‐AML where 16 of 17 IEM‐AML relapses could be identified using the molecular mutations known from initial diagnosis. The highest VAF and mutational burden were observed in the extramedullary tissue (median VAF 45%), followed by cfDNA (median VAF 0.8%, 62% of mutations from IEM‐AML detected), and with lowest levels in mononuclear cells of BM and PB (median VAF 0.05%, 27% of mutations from IEM‐AML detected). The high concordance in mutations between initial diagnosis and IEM‐AML relapse indicates that IEM‐AML relapse after alloHCT represents recipient‐derived relapse, rather than a donor‐derived IEM‐AML post‐alloHCT. Consistent with findings in medullary AML relapse,^38^, ^39^, ^40^ epigenetic modifier mutations were relatively stable and appeared as reliable MRD markers post‐alloHCT, whereas signaling mutations were frequently gained or lost.

Only one *TP53* mutation was detected in the tumor tissue of the 19 IEM‐AML episodes at a limit of detection of 5%, despite four different patients presenting with a complex karyotype at the initial diagnosis. Notably, cytogenetic analysis was not conducted on the tumor tissue. Further chromosomal *TP53* aberrations and subclonal *TP53* mutations cannot be ruled out.

MRD was detectable earlier and more frequently in cfDNA than in BM or PBMC, suggesting that MRD monitoring in plasma provides additional sensitivity for detecting IEM‐AML relapses. In a previous retrospective study of 74 patients who relapsed after alloHCT, we demonstrated that 10%, 38%, and 64% of patients became MRD‐positive at 6, 3, and 1 months before relapse, respectively.^38^ Consistent with these findings, 5 out of 8 patients (62.5%) with available samples within 10 months before IEM‐AML relapse were MRD‐positive, with three of these cases being MRD‐positive only in cfDNA.

NGS‐MRD diagnostics have been evaluated in the context of alloHCT,^30^, ^31^, ^38^, ^41^ and the utility of cfDNA for post‐alloHCT monitoring, along with its prognostic value, has recently been validated. These studies demonstrate that cfDNA offers comparable or even superior sensitivity to BMMC‐based MRD monitoring, particularly in detecting small clones.^17^, ^21^, ^23^, ^42^ While further validation is needed, cfDNA presents a feasible and promising modality for MRD monitoring. In our study, cfDNA proved valuable by detecting IEM‐AML, providing a broader diagnostic scope than BM or PBMC‐based approaches. Furthermore, cfDNA is particularly useful in situations where biopsies pose an unacceptably high risk or where tissue DNA quality is inadequate for analysis, as it enables the identification of targetable mutations. Genomic testing through liquid biopsies plays a crucial role in detecting targetable lesions in solid tumors,^43^, ^44^, ^45^, ^46^ and this approach is equally relevant for IEM‐AML with the growing availability of targeted therapies, such as FLT3‐inhibitors, IDH1‐ and IDH2‐inhibitors, and Menin‐inhibitors.^47^ In the context of early detection post‐alloHCT, cfDNA‐based MRD monitoring has the potential to detect IEM‐AML before clinical manifestations appear.

Imaging and cfDNA analysis complement each other in the management of IEM‐AML. While cfDNA provides valuable information on detecting mutations, imaging remains indispensable for determining tumor extent, guiding histological confirmation through biopsy, and planning therapy, including local treatments such as radiation therapy. Recently, it has been demonstrated that PET/CT can detect EM‐AML lesions that would otherwise remain undiagnosed and has been used for monitoring treatment response during follow‐up.^48^ These findings support the use of PET/CT for both diagnosis and follow‐up in patients with extramedullary involvement. Nevertheless, routine imaging post‐alloHCT for all patients would not be reasonable, as IEM‐AML is a rare complication. In our cohort, the limited use of PET/CT may have contributed to an underestimation of IEM‐AML lesions, as CT and especially MRI often do not provide whole‐body coverage, potentially leading to missed lesions. Additionally, reliance on limited imaging or clinical examination during follow‐up may have further resulted in undetected lesions.

It remains uncertain whether IEM‐AML and EM‐AML, alongside medullary AML, share similar mutational patterns and clonal evolution. Further research is needed to explore this relationship and its implications for detecting extramedullary AML using cfDNA. A recent study investigating newly diagnosed AML patients found no significant differences in mutation profiles between EM‐AML and IEM‐AML; however, the analysis was limited by the small number of patients included.^4^

Additionally, (I)EM‐AML may involve the CNS. To date, the use of cfDNA for monitoring CNS involvement in IEM‐AML has not been reported. In such cases, cfDNA from cerebrospinal fluid is likely the most appropriate source for analysis. Whether cfDNA from plasma is useful in this subset of patients is unknown and warrants investigation in future studies.

Our study did not identify a correlation between the VAF of mutations in IEM‐AML tissue and plasma. Furthermore, the detection rate showed no significant difference between IEM‐AML episodes with a single lesion and those with multiple lesions. The main reason for this observation is likely that cfDNA is a mix of tumor and non‐tumor DNA and that the VAF in plasma may correlate more with overall tumor mass and factors that lead to tumor cell turnover like antineoplastic treatments. However, we did not quantify tumor cell mass and therefore cannot evaluate this association further.

Another limitation of this study is its retrospective nature, which inherently limits the ability to establish causality and generalize the findings. To better assess the risk for patients who become MRD‐positive exclusively in plasma or show a notably higher VAF in PB cfDNA compared to BMMC after alloHCT, a prospective analysis is required. As cfDNA analysis may also complement BM assessment in medullary AML,^22^ not all patients who are MRD positive solely in plasma may develop an IEM‐AML, highlighting the need for cautious interpretation of these results. Prospective studies are essential to validate the use of cfDNA for evaluating therapeutic response in IEM‐AML patients and to correlate these findings with imaging techniques, such as PET/CT, which may provide a more comprehensive assessment of treatment efficacy. Furthermore, the retrospective design may introduce selection bias and limit the ability to fully assess the temporal dynamics of cfDNA levels in relation to treatment response. Future research should focus on longitudinal studies to explore the predictive value of cfDNA over time, which could enhance our understanding of IEM‐AML biology and improve clinical decision‐making regarding therapeutic interventions. Molecular MRD monitoring via NGS, as demonstrated in this study, can be standardized with harmonized protocols and external validation, supporting the applicability of cfDNA‐based NGS in multi‐center studies with appropriate validation.^49^, ^50^

In summary, our retrospective work demonstrates that cfDNA analysis can serve as a mutation‐specific biomarker for the detection of IEM‐AML and outperforms conventional analyses from bone marrow or peripheral blood. While previous reports have been limited to single case studies,^20^, ^23^ our cohort represents the largest analysis to date and strongly supports the utility of cfDNA analyzed by highly sensitive NGS, particularly in the post‐alloHCT setting. These findings might have significant implications for improving the prognosis of this severe complication and may contribute to more effective prevention and treatment strategies for IEM‐AML.

## AUTHOR CONTRIBUTIONS

Henri C. Hupe, Clara P. Wienecke, Felicitas Thol, and Michael Heuser designed the study. Henri C. Hupe, Clara P. Wienecke, Stephan Bartels, Elisa Schipper, Jannika Leßmann, Alina Lasch, Maximilian Bader, Razif Gabdoulline, Martin Neugebohren, Elke Dammann, Hans H. Kreipe, Ulrich Lehmann, Anke K. Bergmann, Nataliya Di Donato, Michael Stadler, Matthias Eder, Arnold Ganser, Florian H. Heidel, Felicitas Thol, and Michael Heuser contributed to the collection of clinical and biological data. Henri C. Hupe, Clara P. Wienecke, Razif Gabdoulline, Stephan Bartels, Elisa Schipper, Ulrich Lehmann, Hans H. Kreipe, Felicitas Thol, and Michael Heuser contributed to the analysis of the clinical and biological data. Henri C. Hupe and Michael Heuser performed the statistical analysis, interpreted the data, and wrote the manuscript. All authors read and agreed on the final manuscript.

## CONFLICT OF INTEREST STATEMENT

M. H. declares honoraria from Astellas, Daiichi Sankyo, Janssen, Miltenyi, Otsuka, Qiagen, and Servier; paid consultancy for Abbvie, AvenCell, Ascentage Pharma, Bristol Myers Squibb, Janssen, Jazz Pharmaceuticals, LabDelbert, Novartis, Pfizer, and Servier; and research funding to his institution from Abbvie, Bayer Pharma AG, Jazz Pharmaceuticals, Glycostem, Karyopharm, PinotBio, Servier, and Toray. S. B. received a speaker honorary from ThermoFisher. U. L. declares honoraria and travel support from AstraZeneca, BMS, GSK, Menarini Stemline, Novartis, QuIP, and Servier. F. H. H. served as an advisor for Novartis, CTI, Celgene/BMS, Janssen, Abbvie, GSK, Merck, and AOP and received research funding from Novartis, Celgene/BMS, and CTI. N. D. D. declares research funding to her institution from Illumina. The remaining authors declare no conflict of interest.

## ETHICS STATEMENT

This study was approved by the institutional review board of Hannover Medical School (ethical votes 936/2011 and 3432‐2016). Patients were registered in the AMLSG Biology and Outcome study (NCT01252485) and written informed consent was obtained according to the Declaration of Helsinki. Clinical data were collected from an electronic database at Hannover Medical School.

## FUNDING

This study was supported by grant 16R/2021 to M. H. from DJCLS, and grants 70114189, 70114478, and 70115044 to M. H. from Deutsche Krebshilfe. H. C. H. received a grant from PRACTIS (DFG funded). F. H. H. was supported by grants of the German Research Council DFG (HE6233/8‐1, project number 449291356; HE6233/9‐1, project number 453491106; HE6233/10‐1, project number 505859092 and in part by HE6233/15‐1 and 16‐1, project number 517204983).

## Supporting information

Supporting information.

## Data Availability

Patient‐level data is not publicly available to comply with the data protection regulations. Reasonable requests for original data should be addressed to the corresponding author.
