# Supplementary material for: Cell‐free DNA for detection and monitoring of extramedullary AML relapse
Source: Hemasphere. 2025 Mar 10;9(3):e70097. doi: 10.1002/hem3.70097 (PMC11891922; doi:10.1002/hem3.70097)
Supplement: Supplementary file 1 — Supporting information. [file HEM3-9-e70097-s001.docx]

**Supplemental Material**

**Cell-free DNA for Detection and Monitoring of Extramedullary AML relapse**

Henri C. Hupe^1^, Clara P. Wienecke^1^, Stephan Bartels^2^, Elisa Schipper^2^, Jannika Leßmann^1^, Alina Lasch^1^, Maximilian Bader^1^, Razif Gabdoulline^1^, Martin Neugebohren^1^, Elke Dammann^1^, Hans H. Kreipe^2^, Ulrich Lehmann^2^, Anke K. Bergmann^3^, Nataliya Di Donato^3^, Michael Stadler^1^, Matthias Eder^1^, Arnold Ganser^1^, Florian H. Heidel^1,4,5^, Felicitas Thol^1^ and Michael Heuser^1,6^

^1^Department of Hematology, Hemostasis, Oncology and Stem Cell Transplantation, Hannover Medical School, Hannover, Germany

^2^Institute of Pathology, Hannover Medical School, Hannover, Germany

^3^Department of Human Genetics, Hannover Medical School, Hannover, Germany^.^

^4^ Cellular Therapy Center (CTC), Hannover Medical School, Hannover, Germany

^5^Leibniz Institute on Aging, Fritz-Lipmann-Institute, Jena, Germany

^6^Department of Internal Medicine IV, University Hospital Halle (Saale), Martin-Luther-University Halle-Wittenberg, Halle, Germany

**Supplemental Figures**

**Supplemental Figure S1. Consort diagram of studied patients.**


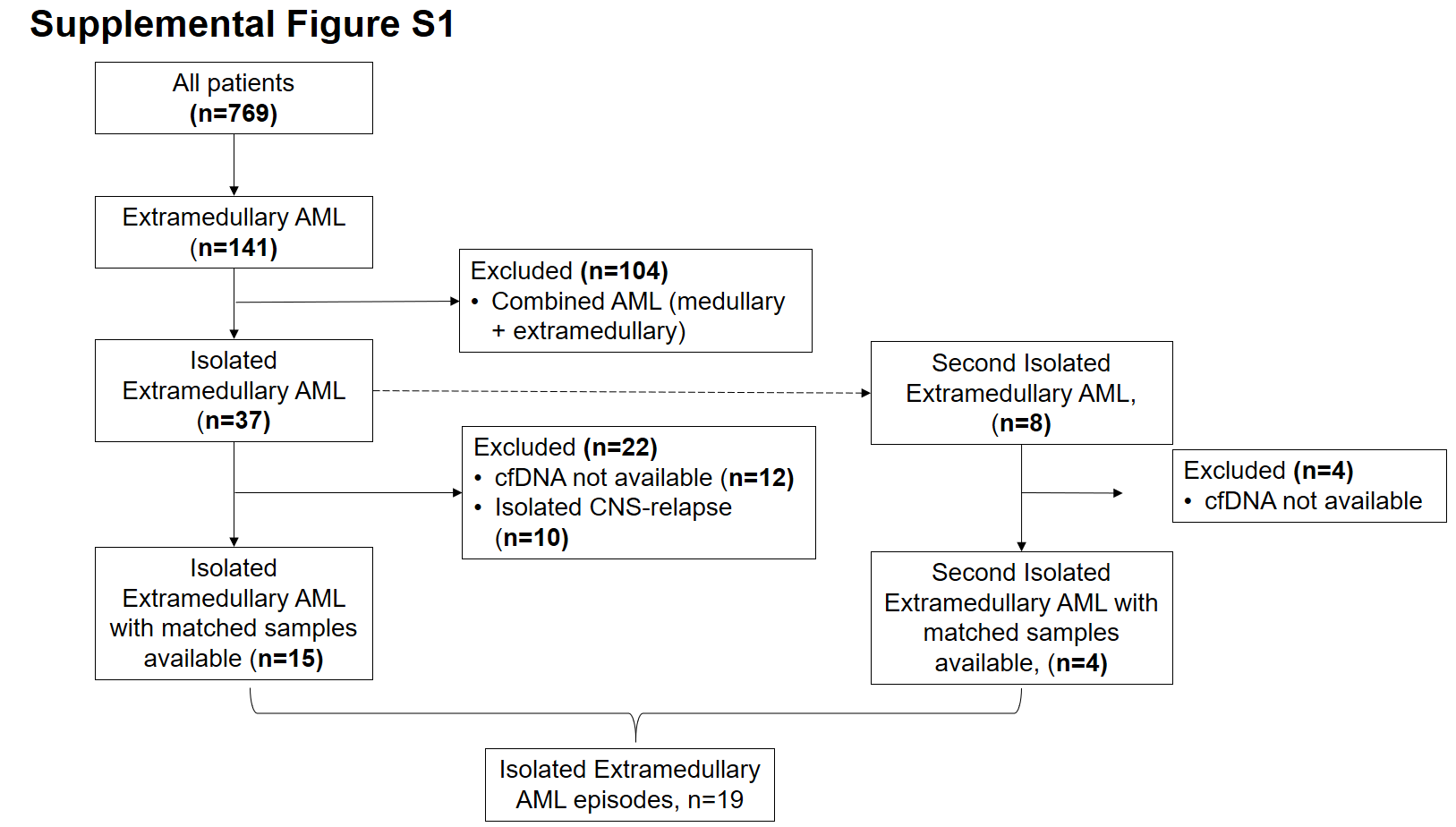


Abbreviations: cfDNA, cell-free DNA; CNS, central nervous system.

**Supplemental Figure S2. Correlation of VAF between IEM-AML tumor and cfDNA at IEM-AML diagnosis and comparison of VAF in IEM-AML tumor tissue between detected and undetected mutations.**

(**A**) VAF of mutations from IEM-AML tumor tissue (y-axis) compared to cfDNA (x-axis) across 16 IEM-AML episodes. Different colors represent distinct mutation classes. For two second IEM-AML episodes, no tumor tissue was available, and therefore they were not included in this analysis. (**B**) Comparison of VAF in IEM-AML tumor tissue shows a median VAF of 45% for mutations detected in cfDNA and 42% for mutations undetected in cfDNA (p=0.123).

A

B


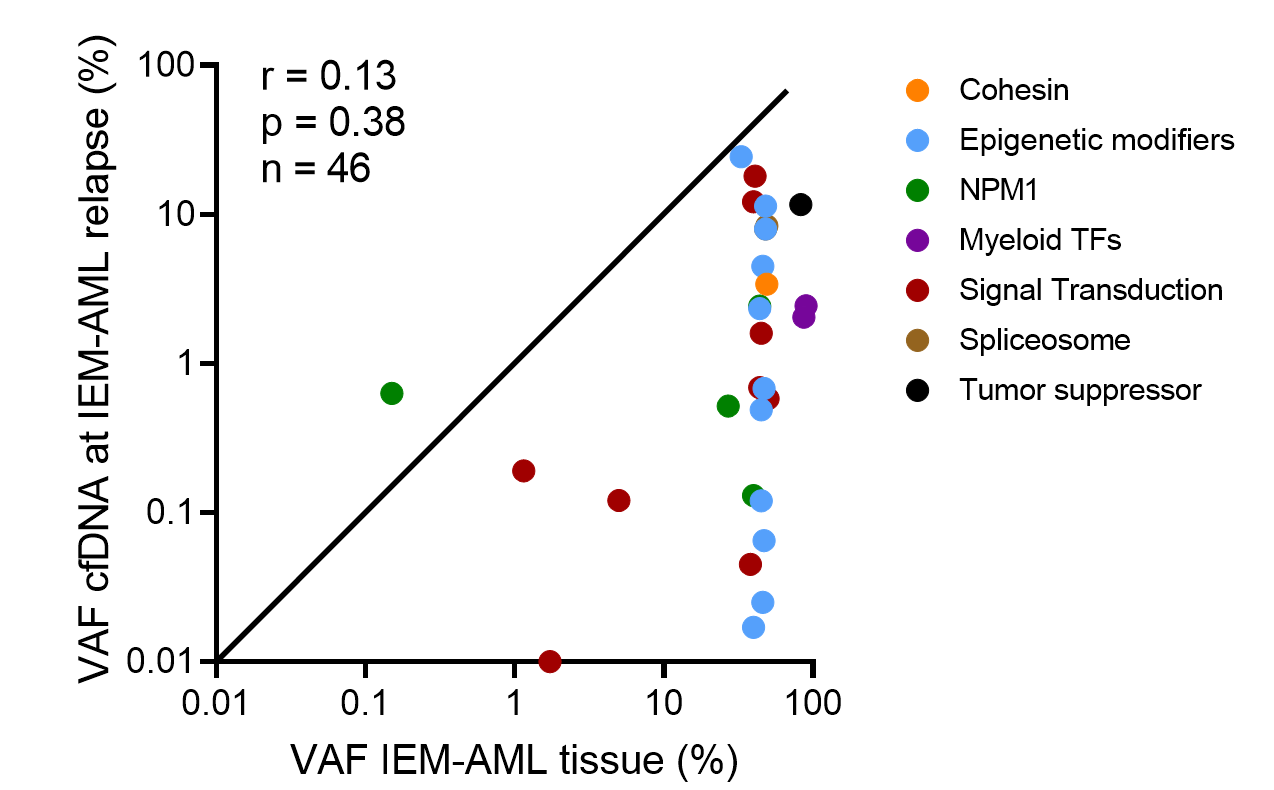


Abbreviations: cfDNA, cell-free DNA; IEM, isolated extramedullary; TFS, transcription factors; VAF, variant allele frequency.

**Supplemental Figure S3. Overlap of mutations between initial diagnosis and IEM AML relapse.**

Number of patients with a specific mutation listed for all mutations detected at initial diagnosis (black) and at the time of IEM-AML relapse(orange).


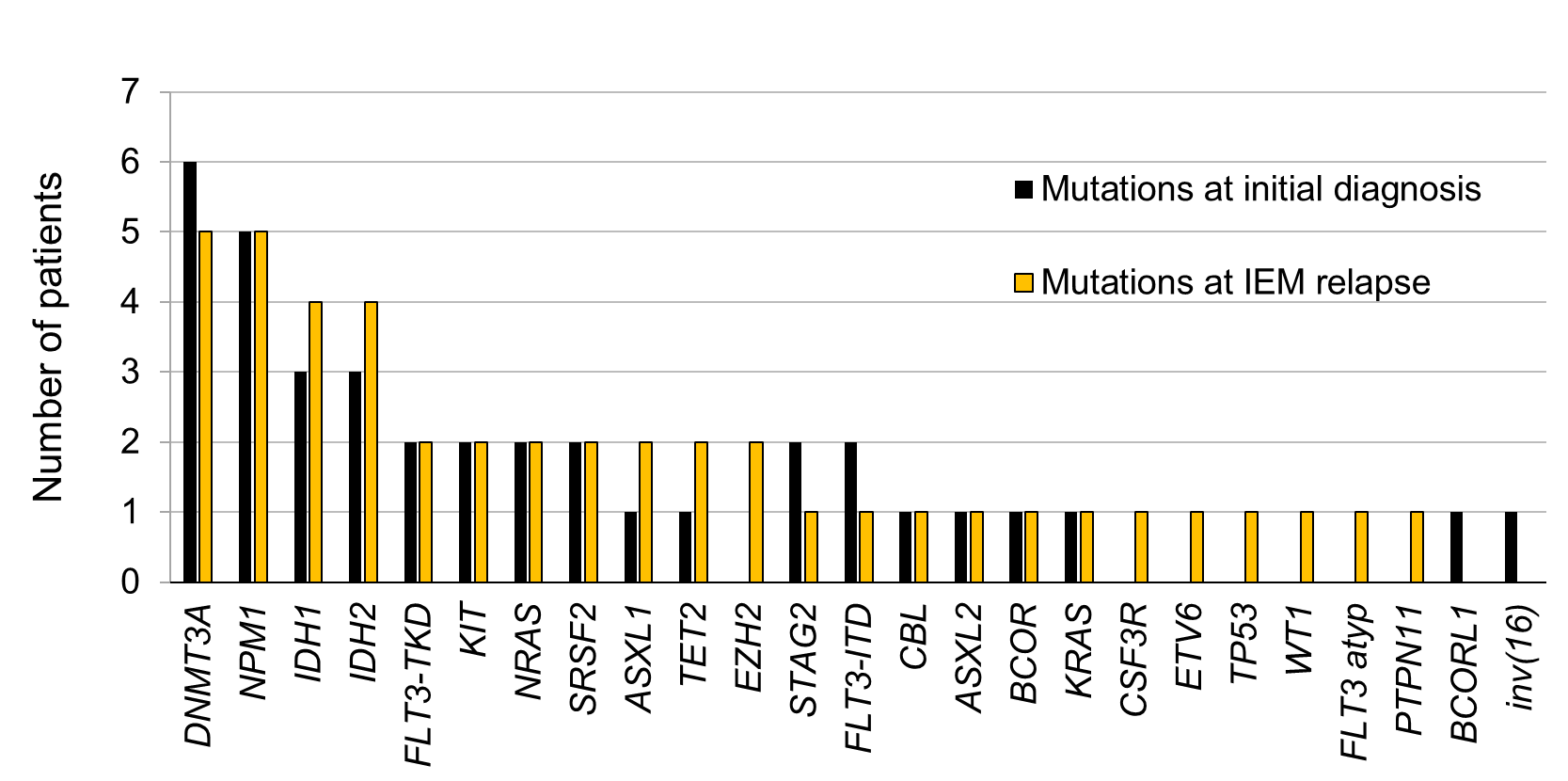


Abbreviations: atyp, atypical; IEM-AML, isolated extramedullary AML; ITD, internal tandem duplication; Inv, inversion**;** TKD, tyrosine kinase domain.

**Supplemental Figure S4. Comparison of cfDNA versus MC-derived DNA for detecting IEM-AML using diagnostic mutations known from initial medullary AML diagnosis.**

**(A)** Number of detected IEM-AML episodes and **(B)** number of IEM-AML mutations in cfDNA and BM/PBMC compartments. One patient with IEM-AML at initial diagnosis and one patient without a molecular marker had to be excluded from this analysis.


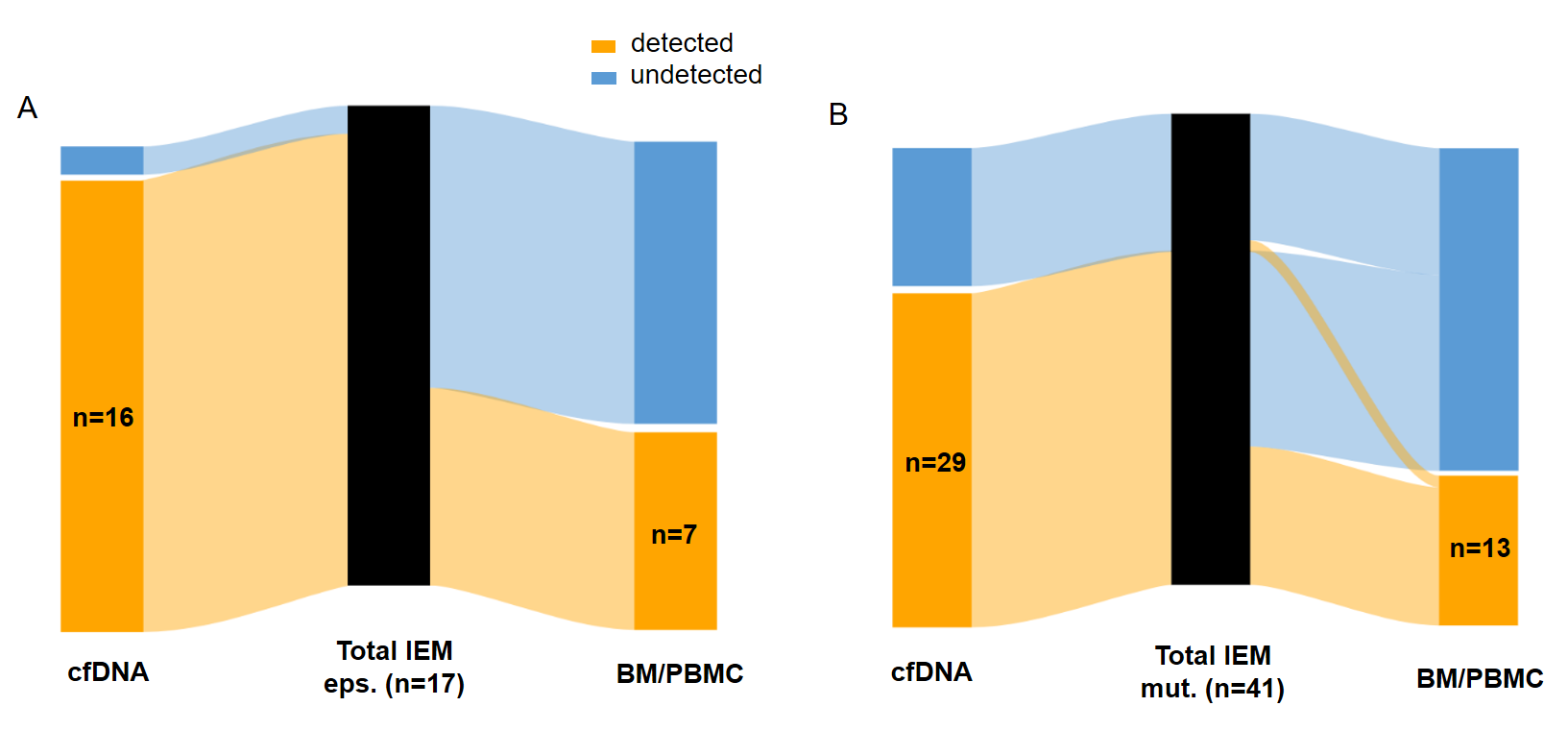


Abbreviations: BM, bone marrow, cfDNA, cell-free DNA; eps, episodes; IEM, isolated extramedullary; MC, mononuclear cells; mut, mutations, PB, peripheral blood.

**Supplemental Figure S5. Comparison of detection rate and VAF between stable and gained mutations at IEM-AML tumor tissue.**

**(A)** Detection rate in cfDNA of stable (71%) versus gained (42%) mutations at IEM-AML relapse. **(B)** VAF of mutations in cfDNA between stable (median VAF 3.95%) and gained (median VAF 1.24%) mutations at IEM-AML relapse (p=ns).


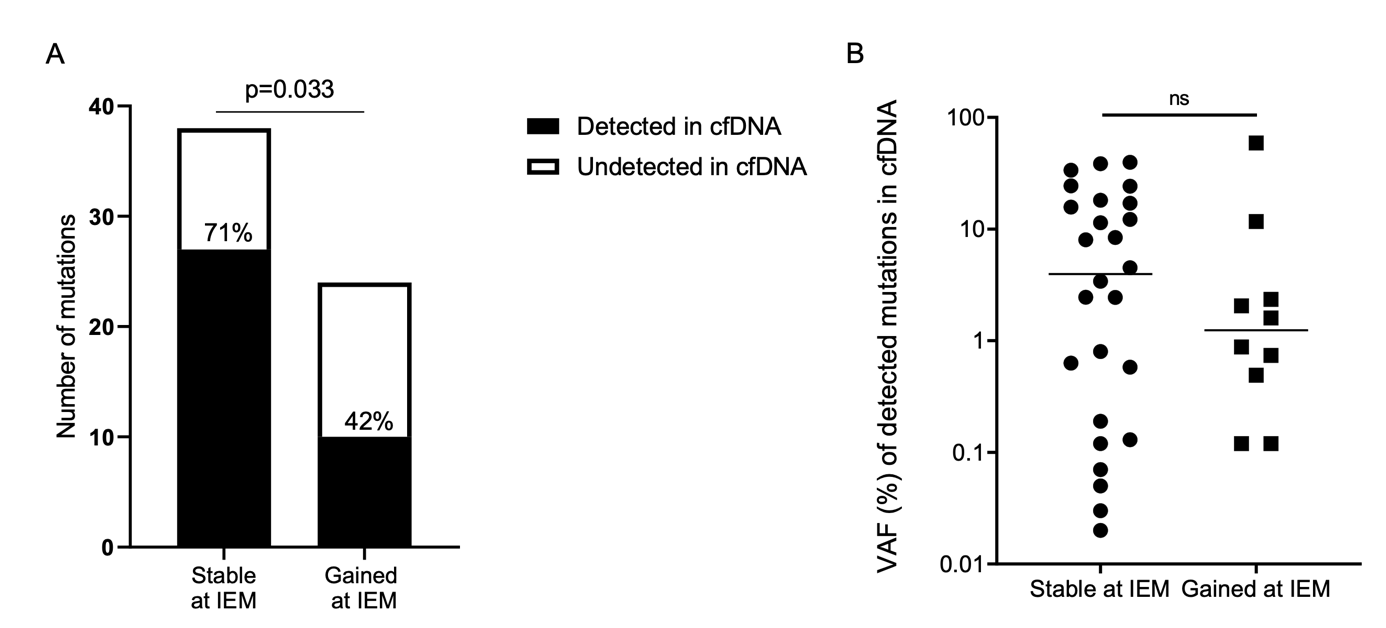


Abbreviations: cfDNA, cell-free DNA; IEM, isolated extramedullary AML; TFS, transcription factors; VAF, variant allele frequency.

**Supplemental Figure S6**. **Clinical and molecular course of the 15 IEM-AML patients.**

The VAFs of all mutations are correlated with the clinical course, including therapies for each individual patient. The red line represents the limit of detection, which is set at 0.01%. The VAFs from cfDNA is shown in orange, while the VAFs from PB/BMMC are represented in blue. The intersection of the X and Y axes marks the first IEM-AML episode. Cellular therapies (alloHCT and DLIs) are indicated by vertical lines.

Abbreviations: AraC, Cytarabine; Aza/Ven, Azacitidine / Venetoclax; CAV, Cladribine, AraC, Venetoclax; cCR, clinically complete remission; cPD, clinically progressive disease; cPR, clinically partial remission; DA, Daunorubicin and Cytarabine; DLI, Donor Lymphocyte Infusion; FLAVIDA, Fludarabin, Cytarabine, Idarubicin, Venetoclax; FLT3-Inh., FLT3-Inhibitor; HAM, High-dose Cytarabine, Mitoxantrone; HD-AraC, high-dose Cytarabine; IAC, Idarubicin, AraC, Cladribine; LDAC, low-dose Cytarabine; LOD, Limit of Detection; NGS, Next-Generation Sequencing; rCR, radiologically complete remission; rPD, radiologically progressive disease; rPR, radiological partial remission; RTX, radiotherapy; VAF, Variant Allele Frequency; Ven, Venetoclax.

**Supplemental Figure S7. Comparison of detection rates across organ involvements**.

Detection rates of mutations in cfDNA (orange bars) and BM/PBMC (blue bars) across various organ involvements. Total number of mutations in the IEM-AML tissue detected by NGS for each organ system are represented by the total bar height. The table below the figure shows the number of detected IEM-AML episodes for each organ system. cfDNA demonstrated consistently higher detection rates across all organ involvements compared to BM/PBMC analysis. The detection rate for cfDNA compared to BM/PBMC was 67% vs. 19% for skin/mucosa and 77% vs. 8% for kidney. For orbital involvement, the rates were 50% vs. 17%, for musculoskeletal involvement 85% vs. 50%, and for lymphatic infiltration 75% vs. 38%. Bladder, mammary tissue, and anterior mediastinal involvement had too few cases for evaluation.


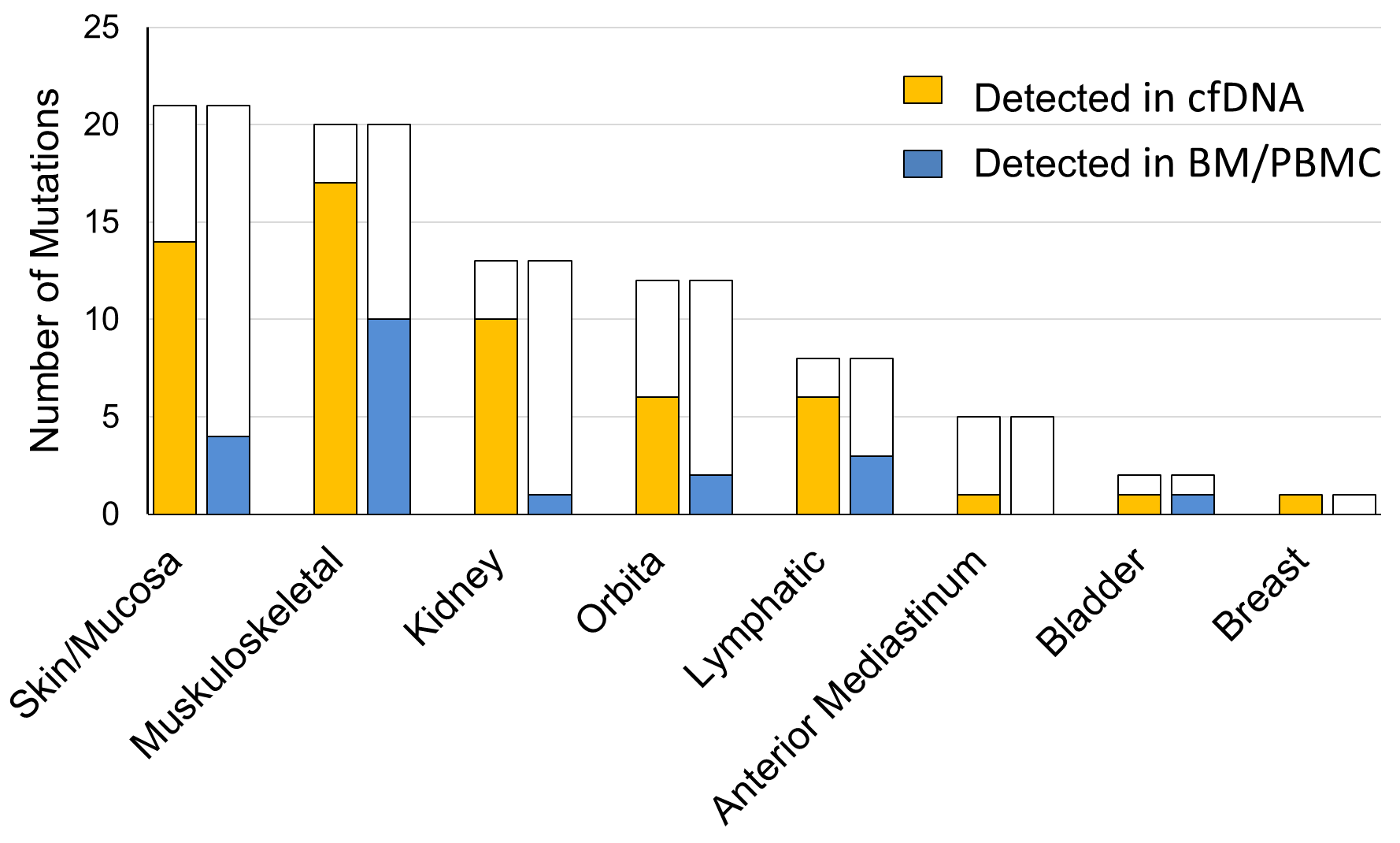


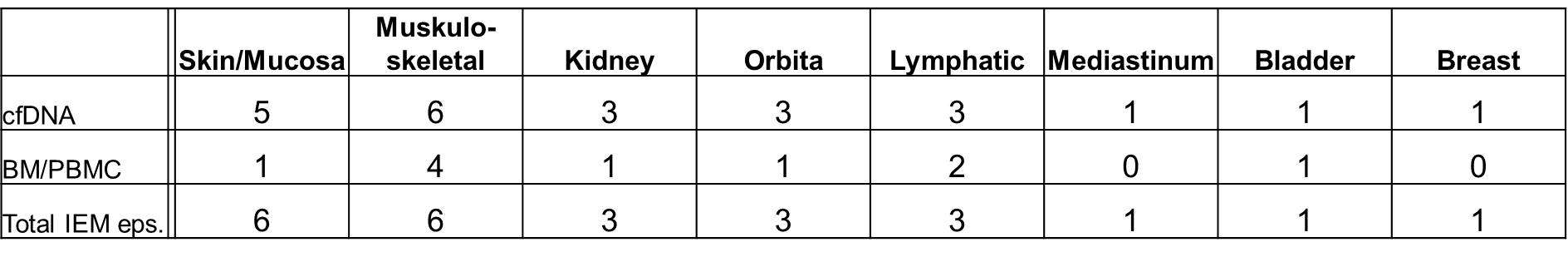


Abbreviations: BM, bone marrow; cfDNA, cell-free DNA; eps, episodes; IEM, isolated extramedullary; MC, mononuclear cells; NA, not available; PB, peripheral blood.

**Supplemental Tables**

**Supplemental Table S1. Genes included in our custom myeloid panel (based on GRCh37/hg19)**

| **Gene** | **Exons** | **Gene** | **Exons** | **Gene** | **Exons** |
| --- | --- | --- | --- | --- | --- |
| *ASXL1* | 12 | *GATA2* | 2-6 | *RUNX1* | complete |
| *ASXL2* | 11+12 | *IDH1* | 4 | *SETBP1* | 4 |
| *BCOR* | complete | *IDH2* | 4 | *SF3B1* | 13-16 |
| *BCORL1* | complete | *JAK2* | 12, 14 | *SMC1A* | 2, 11, 16, 17 |
| *BRAF* | Exon15 | *KDM6A* | complete | *SMC3* | 10, 13, 19, 23, 25, 28 |
| *CALR* | 9 | *KIT* | 2, 8-11, 13, 17 | *SRSF2* | 1 |
| *CBL* | 8, 9 | *KRAS* | 2-5 | *STAG1* | complete |
| *CEBPA* | complete | *MPL* | 10 | *STAG2* | complete |
| *CSF3R* | 14-17 | *MYC* | 2 | *TET2* | 3-11 |
| *CSNK1A1* | 3, 4 | *NF1* | complete | *TP53* | 2-11 |
| *DDX41* | complete | *NPM1* | 12 | *U2AF1* | 2, 6 |
| *DNMT3A* | complete | *NRAS* | 2-5 | *WT1* | 7, 9 |
| *ETNK1* | 3 | *PHF6* | complete | *ZBTB7A* | 2, 3 |
| *ETV6* | complete | *PPM1D* | 1-6 | *ZRSR2* | complete |
| *EZH2* | complete | *PTPN11* | 3, 13 |  |  |
| *FLT3* | 14-16, 20 | *RAD21* | complete |  |  |

**Supplemental Table S2. Overview of mutations detected in IEM-AML tumor at the cDNA level.**

| **Patient** | **Gene used for NGS assessment** | **Mutation at cDNA level** |
| --- | --- | --- |
| 1 | *DNMT3A* | NM_022552.4:c.2644C>T |
| 1 | *IDH1* | NM_005896.4:c.394C>T |
| 1 | *FLT3* | NM_004119.2:c.2039C>T |
| 1 | *EZH2* | NM_004456.4:c.1876G>A |
| 2 | *ASXL2* | NM_018263.4:c.1640A>G |
| 2 | *FLT3* | NM_004119.2:c.2508_2510delCAT |
| 2 | *NRAS* | NM_002524.4:c.35G>A |
| 3 | *KIT* | NM_000222.3: c.2447A>T |
| 4 | *NPM1* | NM_002520.6:c.863_864insTCTG |
| 4 | *IDH2* | NM_002168.4:c.419G>A |
| 4 | *FLT3* | NM_004119.3:c.2503G>T |
| 4 | *DNMT3A* | NM_022552.5:c.2645G>A |
| 5 | *EZH2* | NM_004456.5:c.1753T>C |
| 5 | *IDH1* | NM_005896.4:c.395G>A |
| 5 | *NPM1* | NM_002520.7:c.863_864insCCTG |
| 5 | *FLT3* | NM_004119.3:c.1770_1793dup |
| 5 | *STAG2* | NM_001042749.2:c.1988A>G |
| 6 | *DNMT3A* | NM_022552.5:c.1462C>T |
| 6 | *CSF3R* | NM_156039.3:c.1853C>T |
| 6 | *IDH1* | NM_005896.4:c.395G>A |
| 6 | *NPM1* | NM_002520.7:c.863_864insCATG |
| 6 | *WT1* | NM_024426.6:c.1405G>A |
| 6 | *PTPN11* | NM_002834.5:c.218C>T |
| 7 | *KIT* | NM_000222.3:c.2447A>T |
| 7 | *TET2* | NM_001127208.3:c.3025C>T |
| 7 | *ASXL1* | NM_015338.6:c.1782C>A |
| 8 | *ASXL1* | NM_015338.6:c.1378G>A |
| 8 | *CBL* | NM_005188.3:c.1151G>A |
| 8 | *NPM1* | NM_002520.6:c.859_860insTCTG |
| 9 | *FLT3* | NM_004119.3:c.2503G>T |
| 9 | *RUNX1* | NM_001754.5:c.506_508+3delinsCAGAAC |
| 9 | *CBL* | NM_005188.3:c.1268T>G |
| 10 | *KRAS* | NM_033360.3:c.35G>A |
| 10 | *NPM1* | NM_002520.6:c.860_863dup |
| 10 | *DNMT3A* | NM_022552.4:c.2644C>T |
| 10 | *TET2* | NM_001127208.2:c.651del |
| 11 | *IDH2* | NM_002168.4:c.419G>A |
| 12 | *NRAS* | NM_002524.5:Exon3:c.181C>A |
| 12 | *IDH1* | NM_005896.4:Exon4:c.394C>T |
| 12 | *TP53* | NM_000546.6:Exon5:c.538G>A |
| 12 | *SRSF2* | NM_003016.4:Exon1:c.284C>G |
| 13 | *ETV6* | NM_001987.5:Exon6:c.1082A>G |
| 14 | *FLT3* | NM_004119.3:Exon20:c.2503G>C |
| 14 | *SRSF2* | NM_003016.4:Exon1:c.284_307del |
| 14 | *IDH2* | NM_002168.4:c.419G>A |
| 15 | *DNMT3A* | NM_022552.5:Exon23:c.2645G>A |
| 15 | *NRAS* | NM_002524.5:Exon3:c.181C>A |
| 15 | *IDH2* | NM_002168.4:Exon4:c.514A>T |
| 15 | *BCOR* | NM_001123385.2:Exon4:c.1687_1688insAGAGA |

**Supplemental Table S3.** Overview of diagnostic modalities at IEM-AML manifestation and during the first follow-up within 6 months post IEM-AML.

| **Diagnostic modalities** | |  |
| --- | --- | --- |
| **At 1^st^ IEM-AML** | | n=15 IEM-AML episodes |
| CT |  | 8 (53%) |
| MRI |  | 5 (33%) |
| PET/CT |  | 2 (14%) |
| **At 2^nd^ IEM-AML** | | n=4 IEM-AML episodes |
| CT |  | 1 (25%) |
| PET/CT |  | 1 (25%) |
| Ultrasound | | 1 (25%) |
| Clinical |  | 1 (25%) |
| **At Follow-up (within 6 months post IEM-AML)** |  | n=19 IEM-AML episodes |
| Clinical |  | 8 (42%) |
| CT |  | 3 (16%) |
| PET/CT |  | 3 (16%) |
| MRI |  | 2 (10.5%) |
| Ultrasound |  | 1 (5%) |
| NA early death (within 3 months post IEM-AML) |  | 2 (10.5%) |

Abbreviations: IEM, isolated extramedullary; NA, not available.

**Supplemental Table S4. Cytogenetic analysis at diagnosis and at IEM-AML relapse for each patient.** Cytogenetic analysis was conducted from BMMC.

|  | **At diagnosis (n=15)** | **At 1st IEM-AML relapse (n=9)** | | **At 2nd IEM-AML relapse (n=2)** | |
| --- | --- | --- | --- | --- | --- |
| Patient 1 | Deletion 5q, Tetrasomy 5, 7, 11, | Normal karyotype | |  | |
| Patient 2 | Inversion 16 | Normal karyotype | |  | |
| Patient 3 | Normal karyotype | NA |  |  |  |
| Patient 4 | Normal karyotype | NA |  |  |  |
| Patient 5 | Normal karyotype | Normal karyotype | | Normal karyotype | |
| Patient 6 | Normal karyotype | Normal karyotype | | NA | |
| Patient 7 | Deletion 12p, aberrations involving chromosomes 5, 10, and 14^a^ | NA | |  | |
| Patient 8 | Normal karyotype | Normal karyotype | |  | |
| Patient 9 | Normal karyotype | Normal karyotype | | NA |  |
| Patient 10 | Deletion 9q | NA | |  | |
| Patient 11 | Normal karyotype | Unbalanced  Translocation (7;16),  Isochromosome 17^b^ | |  | |
| Patient 12 | Trisomy 8 | Normal karyotype | | Normal karyotype | |
| Patient 13 | Deletion 5q, Deletion 6p, Deletion 12p, Monosomy 19, Trisomy 20 | NA |  |  |  |
| Patient 14 | Loss of Y, Monosomy 12, Deletion 17p | Normal karyotype | |  | |
| Patient 15 | Normal karyotype | NA |  |  |  |

^a^ external report: details for chromosomes 5, 10, and 14 not fully specified.

^b^ detected in 2 of 25 metaphases ^.^

Abbreviations: IEM, isolated extramedullary; NA, not available.
